# Canadian High School Rugby Coaches Readiness for an Injury Prevention Strategy Implementation: Evaluating a Train-the-Coach Workshop

**DOI:** 10.3389/fspor.2021.672603

**Published:** 2021-05-31

**Authors:** Isla J. Shill, Anu Räisänen, Amanda M. Black, Craig Barden, Carla van den Berg, Carly D. McKay, Stephen W. West, Kati Pasanen, Brent E. Hagel, Carolyn A. Emery

**Affiliations:** ^1^Faculty of Kinesiology, Sport Injury Prevention Research Centre, University of Calgary, Calgary, AB, Canada; ^2^Hotchkiss Brain Institute, Cumming School of Medicine University of Calgary, Calgary, AB, Canada; ^3^Department of Physical Therapy Education, College of Health Sciences, Western University of Health Sciences, Lebanon, OR, United States; ^4^Alberta Children's Hospital Research Institute, University of Calgary, Calgary, AB, Canada; ^5^O'Brien Institute for Public Health, Cumming School of Medicine, University of Calgary, Calgary, AB, Canada; ^6^Department for Health, Centre for Health and Injury and Illness Prevention in Sport, University of Bath, Bath, United Kingdom; ^7^McCaig Institute for Bone and Joint Health, Cumming School of Medicine, University of Calgary, Calgary, AB, Canada; ^8^Tampere Research Center of Sports Medicine, Urho Kaleva Kekkonen Institute, Tampere, Finland; ^9^Departments of Paediatrics and Community Health Sciences, Cumming School of Medicine, University of Calgary, Calgary, AB, Canada

**Keywords:** rugby, coach, workshop, neuromuscular training warm-up, behavior change, intention

## Abstract

**Background:** Canadian rugby coach injury prevention beliefs and attitudes have not been studied, yet are key to informing injury prevention strategy implementation. Despite neuromuscular training (NMT) warm-up success in reducing injury, adoption of these programs is variable. Therefore, objectives of this study included (1) describing Canadian youth rugby coach injury prevention beliefs and attitudes and current warm-up practices and (2) evaluating intention to use a rugby-specific NMT warm-up.

**Methods:** High school rugby coaches completed a questionnaire before and after a rugby-specific NMT warm-up workshop. The pre-workshop questionnaire captured demographics, current warm-up practice, and NMT warm-up knowledge and use. Both questionnaires captured injury prevention beliefs, attitudes and behavioral intention.

**Results:** Forty-eight coaches participated in the workshops. Pre-workshop, 27% of coaches were aware of NMT warm-ups. Coaches primarily included aerobic and stretching components, while balance components were not common in their warm-ups over the past year. Additionally, 92% of coaches agreed to some extent they would “complete a rugby-specific warm-up program prior to every game and training session this season.” Post-workshop, 86% of coaches agreed to some extent that they would use the program in every rugby session. No differences were observed between pre- and post-workshop intention to implement the warm-up (*p* = 0.10).

**Interpretation:** This is the first study to examine current Canadian youth rugby coach warm-up practices and intention to use NMT warm-ups. Canadian rugby coach intention to use a rugby-specific NMT warm-up is high, providing ample opportunity to investigate the efficacy of a NMT warm-up in youth rugby.

## Introduction

Youth rugby union (hereafter “rugby”) participation rates worldwide have increased significantly over the past decade (World Rugby, 2018). Rugby Canada has reported an increase in player registration of 44% between 2012 and 2019 with youth players having a 13% increase (Rugby Canada, 2013, 2020). With the province of Alberta recording the second highest absolute junior rugby registrants in Canada in 2019 (Rugby Canada, 2020). Despite this increased interest, the governing body of high school athletics in Nova Scotia attempted to remove rugby from high schools due to safety concerns (Bradley, 2019). Importantly, few studies have focused on youth rugby in Canada. Injury rates in youth rugby (age 14–18 years) range from 28 to 35 injuries/1,000 match-hours based on a 24 h time-loss definition within countries, such as New Zealand, England, Australia, and Northern Ireland (Leahy et al., 2019). However, the Canadian youth rugby context can differ from that of other countries given players do not typically get their first exposure until high school (age 14–18), there is a shorter playing season, and the playing levels can differ, warranting Canadian-specific youth rugby research to be completed.

Attention has been given to coach education across team sports to facilitate injury prevention strategy implementation. While the literature surrounding rugby coaches is limited, 89% of netball coaches and 96% of soccer and netball coaches reported that they altered the way they coached following a coach education workshop, with most changes related to warm-up/cool-down and stretching protocols (Gianotti et al., 2010). One such warm-up strategy is a neuromuscular training (NMT) warm-up (Olsen et al., 2005). NMT warm-ups consist of aerobic, balance, strength and agility exercises and have been shown to reduce the risk of lower extremity injuries and all injuries by over 30% in youth team sports, leading to considerable reductions in healthcare costs within a season of soccer in Alberta, Canada. (Emery et al., 2015; Marshall et al., 2016). A rugby-specific NMT warm-up was evaluated in English rugby schoolboys. A cluster randomized trial with 40 teams (20 per trial arm) was to used to evaluate efficacy of this NMT warm-up on reducing injury rates. It was demonstrated that teams performing the NMT warm-up three or more times per week suffered 39% fewer match injuries than teams completing the NMT warm-up less than three times per week (Hislop et al., 2017).

Several models have been used to understand the implementation context and guide injury prevention strategy initiatives (Gabriel et al., 2019). One model is the Health Action Process Approach (HAPA), which includes two phases: motivation and volition (Schwarzer, 2008). Psychological constructs that make up the motivation phase include risk perception, outcome expectancy, task self-efficacy, and intention (Schwarzer, 2016). Intention predicts behavior change (volition phase) with some psychological constructs (i.e., action self-efficacy, outcome expectancy, risk perception) predicting intention itself to varying degrees (Schwarzer, 2008). Certain constructs, such as task self-efficacy have been found to be stronger predictors of intention than others (McKay et al., 2016). Constructs that make up the volition phase include maintenance and recovery self-efficacy, action and coping planning, and self-monitoring (Schwarzer, 2016). The intention-behavior gap is an identified gap between these two phases, where some individuals with high intention to change their behavior do not ultimately do so (Schwarzer, 2008). Importantly, understanding the constructs that predict intention can help understand why or why not a behavior change has occurred. More specifically, these constructs can help researchers understand why coaches who have high intention to use evidence-based NMT warm-ups with their teams do not take up or adhere to the warm-up.

An evaluation of the Canadian rugby coaching context is important given the growing popularity of the sport and the lack of literature in this population. This will inform future injury prevention strategy implementation in this setting. Therefore, the primary objectives of this study were to describe (1) Canadian youth rugby coaches' current warm-up practices and coach injury prevention beliefs and attitudes, which included risk perception and outcome expectancy toward youth rugby injury prevention, (2) intention to implement a rugby-specific NMT warm-up before a train-the-coach rugby-specific NMT warm-up workshop, and (3) intention, outcome expectancy, and task and maintenance self-efficacy regarding the NMT warm-up following the workshop. An exploratory objective compared coach intention to deliver a rugby-specific NMT warm-up before and after the workshop.

## Methods

This pre-experimental study was part of the Surveillance in High Schools and Community Sport to Reduce Concussions and their Consequences in Youth (SHRed Concussions), conducted in 16 high schools in Calgary, Canada. All rugby coaches from participating high schools were invited to rugby-specific train-the-coach workshops delivered between January and March 2020, prior to the 2020 high school rugby season (March 2020). Workshop participation was not a requirement to be a part of the larger cohort study. At least one rugby coach attended from 15 of the 16 participating schools. The SHRed Injuries Rugby NMT warm-up coach workshop was developed in partnership with the Sport Injury Prevention Research Centre, Alberta High School Athletics Association, Rugby Canada, and World Rugby, with engagement of community partners, players, coaches, researchers, sport administrators and clinicians in the program development process. Workshops (*N* = 4) took place at the University of Calgary, as well as a high school participating in the SHRed Concussions study. Workshops were administered by two members of the research team (certified exercise physiologist, rugby coach). Pre-workshop questionnaires were administered when coaches arrived for the workshop and post-workshop questionnaires were included immediately following conclusion of the workshop. Participating coaches provided written consent to participate prior to the workshop and pre-workshop questionnaire completion. This study was approved by the University of Calgary Conjoint Health Research Ethics Board (REB 18-2107).

The 2-h workshop was designed and administered considering the HAPA model. Self-efficacy was promoted by coaches practicing teaching and providing one another feedback on program exercises during an active component. Risk perception and outcome expectancy were addressed during an introductory injury prevention presentation and workshop debrief. The workshop structure was designed as such to increase intention to use the NMT warm-up and maximize future adoption of the warm-up. The workshop consisted of a pre-workshop questionnaire (15 min), an injury prevention presentation (15 min), an active component where all exercises were taught and practiced (70 min), a debrief and concluding discussion (15 min), and a post-workshop questionnaire (5 min). Questionnaires were administered electronically, where paper copies were used as a backup in case of technological issues. Paper copies were used for one of the four workshops administered.

The primary purpose of the workshop coach surveys was descriptive and an a-priori sample size was not calculated. Rather, this was in alignment with the sample for a larger quasi-experimental evaluation study to examine the effectiveness of a NMT warm-up in youth rugby players. After completion of the workshop, an email was sent to participating coaches with SHRed Injuries Rugby NMT warm-up resources (i.e., summary handouts, exercise videos, instruction cue cards) to facilitate the implementation of the rugby-specific NMT warm-up. Coaches were made aware they would be receiving these resources during the introductory injury prevention presentation.

Coach injury prevention beliefs and attitudes were measured using HAPA constructs captured during pre- and post-workshop questionnaires (i.e., self-efficacy, risk perception, outcome expectancy, intention). The pre-workshop questionnaire was developed and face validated by researchers from the University of Bath (PI C McKay) and subsequently adapted to include language and demographics relevant to Canadian rugby (e.g., age group, level of play) (PI C Emery). It included four sections: (1) coach demographics, (2) injury perceptions (e.g., injury seriousness, risk of injury), (3) injury prevention attitudes (e.g., injury preventabilility, intention to use a rugby-specific NMT warm-up), and (4) current warm-up practice, NMT warm-up knowledge and use. Coaches were asked to rate their agreement with statements on a 7-point Likert scale (1: Strongly Disagree−7: Strongly Agree). The post-workshop questionnaire was developed by researchers at the Sport Injury Prevention Research Centre and underwent face validation with five individuals familiar with NMT warm-ups (i.e., a community partner representative, physical education teacher, youth rugby coach, personal trainer, high performance sport director). The questionnaire evaluated post-workshop intention to use the NMT warm-up, outcome expectancy with using the NMT warm-up, and task and maintenance self-efficacy for using and implementing the NMT warm-up. A 5-point Likert scale (1: Strongly Disagree−5: Strongly Agree) was used to record coaches' agreement with the statements. Two different Likert scales were used as both surveys were in alignment with other projects and to facilitate a cross-site rugby coach analysis.

Statistical analyses were conducted using STATA v16 (StataCorp, 2020). Proportions and means (standard deviations) were calculated to describe coach baseline characteristics. Baseline characteristics that are presented as means were tested for normality. Proportions were used for any characteristics that were categorical data. Medians and interquartile ranges (IQR) were calculated for statements concerning coach injury prevention beliefs and attitudes as statements are ordinal data. In the pre- and post-workshop questionnaires, questions that pertained to a construct from the HAPA model (i.e., risk perception, outcome expectancy, intention, self-efficacy) were grouped as such and an overall median and interquartile range (IQR) was calculated for each construct. If only one question was used to assess a HAPA construct, the median and IQR of that question was taken as a representation of the construct. Questions that had missing reponses were reported as missing. If coaches only completed the pre-workshop questionnaire, they were excluded from the cross-time point intention analysis.

Given the pre-workshop questionnaire was based on a 7-pt Likert scale and the post-workshop scale was based on a 5-pt Likert scale, exploratory analyses considered two different methods to evaluate the difference between intention across the two timepoints. Two different conversion methods were used to perform a sensitivity analysis to understand if a change would be detected given the limitations surrounding the use of different Likert scales. The conversion methods were facilitated by the lack of variability within intention scores and a clustering of results at the extremity of the Likert scale at both timepoints. Intention scores were converted firstly by collapsing both pre-workshop intention (“I would like my team to complete a rugby-specific warm-up program prior to every game and training session next season”) and post-workshop intention (“I will conduct the SHRed Injuries program in every session with my athletes”) into a 3-point Likert scale (i.e., agree, neither agree nor disagree, disagree). The second method included collapsing the pre-workshop intention score to a 5-pt Likert scale to match the pre-existing post-workshop intention scale. Once the conversions were completed, two separate Wilcoxon signed rank tests were used to compare intention. Statistical significance was set at α = 0.05.

## Results

Forty-eight coaches completed questionnaires (45 completed pre- and post-workshop; three completed pre-workshop only). There were no dropouts during completion of the questionnaires; however, three coaches were unable to complete the post-workshop questionnaire, having left the workshop before completion. Preworkshop response rate was 48/48 (100%). Postworkshop response rate was 45/48 (94%). Coach characteristics are presented in Table 1.

**Table 1:** Coach characteristics.

**Characteristics**		**Total (*n* = 48) (%)**	**Missing**
Age, mean (SD)		38.5 (11)	10
Sex, *n* (%)	Male	34 (71)	0
	Female	14 (29)	
Previous playing experience, *n* (%)	Yes	33 (69)	0
	No	15 (31)	
Coaching role	Athletics director	1 (2)	1
	Head coach	21 (45)	
	Assistant coach	20 (43)	
	Team manager	1 (2)	
	Other	4 (9)	
Years coaching, *n* (%)	Never	6 (13)	0
	<2 years	4 (8)	
	2–3 years	5 (10)	
	4–5 years	10 (21)	
	6+ years	23 (48)	
Coaching certification, *n* (%)	Yes	25 (53)	1
	No	22 (47)	
Previously heard of NMT warm-up, *n* (%)	Yes	13 (27)	0
	No	26 (54)	
	Unsure	9 (19)	

### Previous Warm-Up Practice

**Figure 1:**
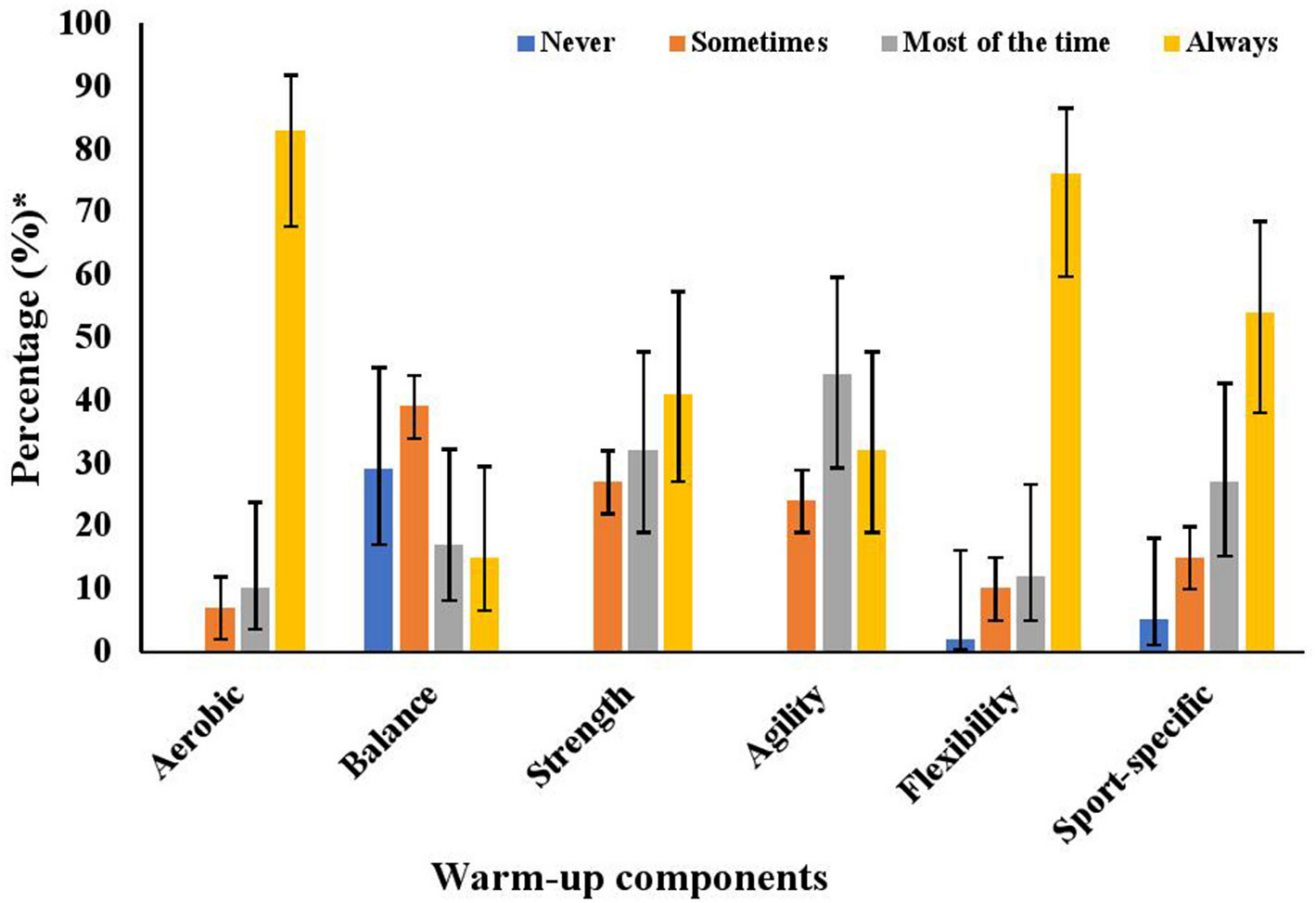
Previous warm-up routine of coaches who reported using a warm-up in the past year. ^*^Whiskers of each bar display the 95% confidence interval of the point estimate.

Eighty-five percent (95% CI: 72–94) of coaches reported using a warm-up with their team within the past 12-months. Warm-up components that coaches most commonly reported always using were aerobic (83%, 95% CI: 68–92) and flexibility (76%, 95% CI: 60–87) (Figure 1). Fifteen percent (95%CI: 6–29) of coaches reported always using balance, while 29% (95%CI: 17–45) reported never using balance (Figure 1). Coaches reported a median warm-up duration of 15 min (IQR: 12.5–20).

### Coach Beliefs and Attitudes: Intention, Risk Perception, Outcome Expectancy, and Self-Efficacy

Coaches rated spinal injury (92%, 95% CI: 79–97) and concussion (79%, 95% CI: 64–88) in the category of “most serious” rugby injuries (Table 2). Bone fracture (38%, 95% CI: 25–53), overuse injury (17%, 95% CI: 8–30), shoulder injury (15%, 95% CI: 7–29) and knee injury (10%, 95% CI: 4–23) were reported by coaches to be “very” serious in severity (Table 2). Coaches perceived cuts/scrapes (44%, 95% CI: 30–58) and bruises/contusions (23%, 95% CI: 13–38) as “not serious” (Table 2). Coaches could utilize each of the seven categories as many times as they perceived necessary.

**Table 2:** Coach injury severity perceptions on pre-workshop questionnaire.

**Injury**	**Frequency (%)**							**Median (IQR)**	**Missing**
	**1: Not serious**	**2**	**3**	**4: Moderately serious**	**5**	**6**	**7: Very serious**		
Spinal Injury	0	0	0	0	0	4 (9)	43 (92)	7 (7–7)	1
Concussion	0	0	0	0	5 (2)	9 (19)	37(79)	7 (7–7)	1
Bone Fracture	0	0	0	7 (15)	11 (23)	11 (23)	18 (38)	6 (5–7)	1
Overuse injury	0	1 (2)	7 (15)	13 (27)	5 (10)	14 (29)	8 (17)	5 (4–6)	0
Shoulder injury	0	0	2 (4)	8 (17)	19 (40)	11 (23)	7 (15)	5 (5–6)	1
Knee	0	1 (2)	3 (6)	13 (27)	17 (35)	9 (19)	5 (10)	5 (4–6)	0
Bruise/contusion	11 (23)	22 (47)	8 (17)	4 (9)	1 (2)	0	1 (2)	2 (1–7)	1
Muscle strain	2 (4)	17 (36)	12 (26)	11 (23)	3 (6)	2 (4)	0	3 (2–4)	1
Ankle	1 (2)	4 (9)	11 (23)	24 (51)	5 (11)	2 (4)	0	4 (3–4)	1
Cut/scrape	21 (44)	18 (38)	6 (13)	1 (2)	2 (4)	0	0	2 (1–2)	0

All responses from the pre-works Supplementary Material 1. Before the workshop, coaches reported high intention to “complete a rugby-specific warm-up prior to every game next season,” with 44 (92%) coaches agreeing or strongly agreeing. Questions that pertained to negative injury outcomes (i.e., negative outcome expectancy) had a median score of 6/7 (IQR: 6–7). Twenty-two (45%) coaches agreed or strongly agreed that they “expect a player they coach to sustain a rugby injury sometime during the next season.” Questions that pertained to positive injury prevention outcomes (i.e., positive outcome expectancy) had a median score of 6/7 (IQR: 6–7) and 45 (94%) coaches agreed or strongly agreed that “it is possible to prevent some rugby injuries.” Coach risk perception was evaluated by two questions. The first question (i.e., “*Injuries are not a problem for my athletes”)* targeted coach risk perception with his/her own team, while the second question (i.e., “*Rugby players are at a high risk of suffering an injury”*) evaluated coach risk perception within rugby broadly. Coach risk perceptions with their team and the sport had median scores of 2/7 (IQR:1–2) and 5/7 (IQR: 4–6), respectively.

All responses from the post-workshop questionnaire can be found in Supplementary Material 2. After the workshop, coach intention to “conduct the SHRed Injuries program in every session with their students/athletes” was high with 85% (95% CI: 58–100) of coaches partly or strongly agreeing. Post-workshop task self-efficacy was high in coaches with a median positive task-self efficacy score of 5/5 (IQR: 4.5–5) and a median negative task self-efficacy score of 1.5/5 (IQR: 1–2). Maintenance self-efficacy was very high with a median score of 5/5 (IQR: 4.5–5). Following the workshop, coaches had high outcome expectancy with 41 (61%, missing 4) coaches strongly agreeing that they “believe using the SHRed Injuries program regularly will reduce the number of injuries in their athletes.” Thirty-six (80%) coaches strongly agreed that their “environment (e.g., school, club) supports the implementation of the SHRed Injuries program.”

Based on exploratory analyses, coach intention to use a rugby-specific NMT warm-up across the two timepoints (i.e., pre-workshop, post-workshop) did not change. No statistically significant relationship was found between the two timepoints using either conversion method (i.e., 3-point Likert scale *p*-value = 0.1005, 5-point Likert scale *p*-value = 0.4001). Pre- and post-workshop intention change is reported in Table 3.

**Table 3:** Pre- and post- “Train-The-Coach” SHRed Injuries workshop scores with Likert scale conversions [frequency (%)].

		**Strongly disagree**	**Disagree**	**Slightly disagree**	**Neither agree nor disagree**	**Slightly agree**	**Agree**	**Strongly agree**
Pre-workshop intention	7-point Likert scale (original)	0	0	0	2 (4)	2 (4)	25 (52)	19 (40)
	5-point Likert scale	**Strongly disagree**	**Disagree/Slightly disagree**		**Neither**	**Agree/Slightly agree**		**Strongly agree**
		0	0		2 (4)	27 (56)		19 (40)
	3-point Likert scale	**Disagree**			**Neither**	**Agree**		
		0			2 (4)	46 (96)		
Post-workshop intention (Missing: 4)	5-point Likert scale (original)	**Strongly disagree**	**Partly disagree**		**Unsure**	**Partly agree**		**Strongly agree**
		0	1 (2)		5 (11)	20 (45)		18 (41)
	3-point Likert scale	**Disagree**			**Neither**	**Agree**		
		1 (2)			5 (11)	9 (86)		

## Discussion

### Previous Warm-Up and Warm-Up Beliefs

Of the coaches that reported using a warm-up routine in the past 12 months, the use of NMT components (i.e., aerobic, balance, strength, agility) within their warm-ups was variable with most coaches always using aerobic components and few using balance. This is similar to findings in Canadian youth basketball, where coaches reported including an aerobic component in their warm-up, but only 27% utilized balance exercises prior to attending a workshop on a NMT warm-up (Raisanen et al., 2021). Within the literature, balance appears to be the most neglected component of coaches' previous warm-ups, though research suggests that it is an effective and important component (Brunner et al., 2019). Previous research has established that balance exercises significantly reduce the risk of ankle injury in high school athletes and improve static and dynamic balance following a balance training program (Emery et al., 2005; McGuine and Keene, 2006). Further, considering other components of a NMT warm-up, strength exercises have been associated with a significant reduction in ACL injuries (Sugimoto et al., 2015). However, adoption of these exercises within a warm-up is limited. Further understanding of the barriers to the use of strength, balance and agility exercises in a warm-up program in youth rugby should be explored in future research. In a school setting, barriers identified may include lack of knowledge of their relevance to injury prevention, complexity and time to complete exercise components, and planning required to carry out a NMT warm-up in its' entirety (Richmond et al., 2020). Considering the use of strength exercises is important given the intense, collision-nature of rugby and the high physical demands of the sport.

Adoption of NMT warm-ups as a coach's standard practice is variable despite high intention to use the warm-up initially (McKay et al., 2016). Previously, significant facilitators for a successful NMT warm-up in a junior high school setting were identified as strength and quality of evidence supporting the warm-up, adaptability of the warm-up, implementation climate, culture, and compatibility of the program with users (Richmond et al., 2020). Within our study, 54% of rugby coaches always include sport-specific components in their warm-up. A facilitator to consider when addressing the implementation context for NMT warm-ups within youth rugby is adjusting the NMT warm-up to be sport-specific, as what was done in the current study, to increase program adoption (Emery et al., 2015).

### Intention

Prior to the workshop, coach intention to administer a rugby-specific warm-up was high. These results are similar to those of previous studies. High intention to implement an ACL injury prevention program prior to a coach workshop was established in elite-level youth soccer coaches (Frank et al., 2015). Importantly, they suggested that efforts should focus on reducing barriers to facilitate adoption and adherence and promote general application of the program given the already high levels of intention in these coaches (Frank et al., 2015).

Despite there being no statistically significant difference, based on exploratory analyses between the two timepoints, it is important to highlight the few coaches that may have had less intention following the workshop (Table 3). McKay et al. (2016) highlight the importance of understanding barriers and facilitators of implementation to improve adoption, as well as future adherence to the program. Future research should evaluate if coaches' feel they have enough support and resources to implement a NMT warm-up. Coach support and resources should target teaching proper exercise technique and how to manage/facilitate the warm-up in their specific setting. The coaches may have had a more realistic expectation regarding NMT warm-up implementation after the workshop. Certain aspects they might not have previously considered prior to the workshop could be the importance of teaching proper exercise technique and how to manage/facilitate the warm-up in their specific setting. Ultimately, these factors could lead to less intention and potentially not adhereing to, or even using the warm-up.

### Risk Perception, Outcome Expectancy, and Task Self-Efficacy

Within the sport injury literature, risk perception has not been shown to be a significant predictor of intention while task self-efficacy and outcome expectancy have (McKay et al., 2016; Zhang et al., 2019). Our results show that Canadian rugby coaches are aware of the risk associated with participating in the sport. Given the two ways risk perception was addressed within the study (i.e., team-specific and general sport level), coaches' personal experiences should be considered. Within a cohort of female youth soccer coaches, years of playing experience was found to be negatively associated with high adherence to a NMT warm-up (McKay et al., 2014). Moreover, a coach's personal experience with rugby, such as previous years of playing experience, could also alter how a coach views rugby broadly and injury outcomes. This could affect their receptiveness to injury prevention strategies for the sport and future adherence, as was the case with youth female soccer coaches.

Regardless of negative or positive outcome expectancy before the workshop, coaches had strong outcome expectancy, such as expecting a rugby-specific warm-up to reduce the risk of injury and improve physical charateristics in their players and expecting one of their players to be injured within the upcoming season. Within the motivational phase, positive outcome expectancy is a more significant predictor of intention than negative outcome expectancy (Schwarzer, 2008). Moreover, task self-efficacy scores at the post-workshop timepoint were high, reflecting the coaches' firm belief in their ability to administer the NMT warm-up. As task-self efficacy has been found to be the strongest predictor of intention (McKay et al., 2016), it is understandable that post-workshop intention to administer the NMT warm-up remained high. Nevertheless, it would be important to understand the ability of the motivational phase constructs (i.e., risk perception, outcome expectancy, task self-efficacy) to predict intention. Further investigation into how a coach's personal experiences (e.g., previous playing and coaching experience, injury history) shape injury prevention beliefs and attitudes would be beneficial to understand behavior change.

Ultimately, the behavior being assessed in this study is the uptake and continued use of this rugby-specific NMT warm-up from the coaches, but follow-up with the coaches could not be completed due to the COVID-19 pandemic cancellation of the 2020 high school rugby season. The present evaluation describes Canadian youth rugby coach injury prevention beliefs, attitudes, and behavioral intention following an injury prevention strategy implementation initiative. The results of this study describe the implementation context in order to evalute the effectiveness of a a rugby-specific NMT warm-up in this population (Schwarzer, 2008).

### Limitations

Due to the nature of the workshops and this being a part of a larger study, coaches self-selected to attend the workshop. Self-selection could inherently result in selection bias and overestimate intention and other injury prevention belief outcomes in this youth rugby coach population. Coaches who selected to come may have had increased interest in the programs and injury prevention, while coaches who did not attend might not have an interest in injury prevention strategies. We had coach representation from 15/16 schools invited to the study, which does limit the potential aforementioned effects of selection bias. As this study used an inclusive sampling strategy, participation was a representative convenience sample across schools. Therefore, generalizability of these findings could be limited. Moreover, confirmation bias could have overestimated the injury prevention beliefs and attitudes outcomes as coaches were attending a workshop on a rugby-specific NMT warm-up; therefore, they might be more likely to say they are intending to use it and be more mindful of injury prevention, which would also overestimate our results.

Two different Likert scales were used at the pre- and post-workshop timepoints as these questionnaires were aligned with other studies at the University of Bath and the Sport Injury Prevention Research Centre. Using different questionnaires was an initial attempt to implement a cross-site comparison with the pre-workshop questionnaires while still being able to compare against other research at the Sport Injury Prevention Research Centre. It is important to note that collapsing Likert scale categories as per the two conversion methods used does limit the amount of variability in our intention outcome. Moreover, when presented with different levels of Likert scales, a participant's response could differ due to the amount of options available to them when responding to a questionnaire (Dolnicar and Grün, 2013). However, intention was very high, resulting in a considerable lack of variability in intention scores, which facilitated a cross timepoint analysis despite the intention scales differing at the two timepoints.

The sample size for this study was small and power in the present study was limited. An assessment for potential modification and confounding of covariables (e.g., previous coaching experience, previous injury, coach qualifications) for intention could not be completed.

## Conclusion

This is the first study to examine NMT warm-ups in the Canadian youth rugby coaching context. Our results suggest that Calgary rugby coaches' intentions to use a rugby-specific NMT warm-up with their respective teams is high, providing opportunity for further investigation into the effects of a NMT warm-up within this population. The findings will be useful to guide future interventions in youth Canadian rugby. Moreover, future research should evaluate how HAPA constructs (i.e., pre-intenders, intenders) can predict coach adherence to a rugby-specific NMT warm-up and further explore the adoption of the NMT warm-up with coaches.

## Data Availability Statement

The datasets presented in this article are not readily available because private medical data. Requests to access the datasets should be directed to isla.shill@ucalgary.ca.

## Ethics Statement

The studies involving human participants were reviewed and approved by University of Calgary Conjoint Health Research Ethics Board. The patients/participants provided their written informed consent to participate in this study.

## Author Contributions

IS, AB, BH, KP, and CE contributed to the study design. CM and CBa constructed the preworkshop questionnaire. AR constructed the postworkshop questionnaire. IS and CBe contributed to conducting the coach workshops and data entry and management. IS conducted data cleaning and analysis. CE was the nominated PI for the larger cohort. All authors critically reviewed and edited the manuscript before submission.

## Conflict of Interest

The authors declare that the research was conducted in the absence of any commercial or financial relationships that could be construed as a potential conflict of interest.
