# Supplementary material for: Canadian High School Rugby Coaches Readiness for an Injury Prevention Strategy Implementation: Evaluating a Train-the-Coach Workshop
Source: Front Sports Act Living. 2021 May 31;3:672603. doi: 10.3389/fspor.2021.672603 (PMC8200819; doi:10.3389/fspor.2021.672603)
Supplement: Supplementary file 1 [file Data_Sheet_1.docx]

Supplementary Material 1. Pre-workshop questionnaire HAPA construct question outcomes

| **Statement** | **Frequency (%)** | | | | | | | **Median (IQR)** | **Missing** |
| --- | --- | --- | --- | --- | --- | --- | --- | --- | --- |
|  | **1:**  **Strongly disagree** | **2:**  **Disagree** | **3:**  **Slightly disagree** | **4:**  **Neither** | **5:**  **Slightly agree** | **6:**  **Agree** | **7:**  **Strongly agree** |  |  |
| **Intention** | | | | | | | | | |
| *I would like my team to complete a rugby specific warm-up program prior to every game and training session this season* | 0 | 0 | 0 | 2 (4) | 2 (4) | 25 (52) | 19 (40) | 6 (6-7) | 0 |
| **Risk Perception** | | | | | | | | | |
| *Injuries are not a problem for my athletes* | 15 (31) | 22 (46) | 5 (10) | 1 (2) | 3 (6) | 1 (2) | 1 (2) | 2 (1-2) | 0 |
| *Rugby players are at a high risk of suffering an injury* | 1 (2) | 4 (8) | 3 (6) | 5 (10) | 15 (31) | 13 (27) | 7 (15) | 5 (4-6) | 0 |
| **Positive Outcome Expectancies** | | | | | | | | | |
| *It is possible to prevent some rugby injuries* | 0 | 0 | 1 (2) | 1 (2) | 1 (2) | 24 (50) | 21 (43) | 6 (6-7) | 0 |
| *Completing a rugby specific warm-up program prior to every game and training session will reduce the risk of players sustaining the injury* | 0 | 1 (2) | 0 | 1 (2) | 7 (15) | 24 (50) | 15 (31) | 6 (6-7) | 0 |
| *Completing a rugby specific warm-up program prior to every game and training session will improve physical characteristics such as balance, agility, and strength* | 0 | 0 | 0 | 1 (2) | 4 (8) | 24 (50) | 19 (40) | 6 (6-7) | 0 |
| *Static stretching can prevent rugby injuries* | 0 | 0 | 1 (2) | 3 (6. | 5 (10) | 31 (65) | 8 (17) | 6 (6-7) | 1 |
| *Controlled jumping/landing exercises can prevent rugby injuries* | 0 | 0 | 0 | 3 (6) | 9 (19) | 28 (58) | 8 (17) | 5.5 (6-7) | 0 |
| *A warm-up jog/run can prevent rugby injuries* | 0 | 0 | 2 (4) | 3 (6) | 10 (21) | 27 (56) | 6 (13) | 6 (5-6) | 0 |
| *Cutting exercises can prevent rugby injuries* | 1 (2) | 6 (13) | 2 (4) | 8 (17) | 8 (17) | 19 (40) | 4 (8) | 5 (4-6) | 0 |
| *Cool-down jog/run can prevent rugby injuries* | 0 | 0 | 2 (4) | 4 (8) | 14 (29) | 24 (50) | 4 (8) | 6 (5-6) | 0 |
| *Resisted shoulder exercises can prevent shoulder injuries in rugby* | 0 | 0 | 1 (2) | 2 (4) | 8 (17) | 25 (52) | 12 (25) | 6 (6-6.5) | 0 |
| *Neck muscle strengthening can prevent concussion in rugby* | 3 (6) | 2 (4) | 4 (8) | 10 (20) | 6 (13) | 12 (25) | 11 (23) | 5 (4-6) | 0 |
| *Trunk (core) strengthening exercises can prevent rugby injuries* | 0 | 0 | 0 | 0 | 10 (20) | 22 (46) | 16 (33) | 6 (6-7) | 0 |
| **Negative Outcome Expectancies** | | | | | | | | | |
| *Rugby injuries can shorten a player's career* | 1 (2) | 2 (4) | 1 (2) | 0 | 5 (10) | 9 (19) | 30 (63) | 7 (6-7) | 0 |
| *Rugby injuries can cause physical problems later in life* | 1 (2) | 1 (2) | 1 (2) | 0 | 5 (10) | 15 (31) | 26 (54) | 7 (6-7) | 0 |
| *Rugby injuries can have a negative impact on team performance* | 5 (10) | 2 (4) | 2 (4) | 2 (4) | 13 (27) | 14 (29) | 12 (25) | 6 (5-6.5) | 0 |
| *Rugby injuries can have a negative impact on a player's quality of life* | 0 | 0 | 6 (13) | 2 (4) | 10 (21) | 17 (35) | 13 (27) | 6 (5-7) | 0 |
| *I expect a player I coach to sustain a rugby injury sometimes during the next season* | 2 (4) | 4 (8) | 4 (8) | 5 (10) | 11 (23) | 12 (25) | 10 (21) | 5 (4-6) | 0 |

Supplemental Material 2. Post-workshop questionnaire HAPA construct question outcomes

| **Statement** | **Frequency (%)** | | | | | **Median (IQR)** | **Missing** |
| --- | --- | --- | --- | --- | --- | --- | --- |
|  | **1:**  **Strongly disagree** | **2:**  **Partly disagree** | **3:**  **Unsure** | **4:**  **Partly agree** | **5:**  **Strongly agree** |  |  |
| **Intention** | | | | | | | |
| *I will conduct the SHRed Injuries program in every session with my students/athletes/clients* | 0 | 1 (2) | 5 (11) | 20 (45) | 18 (41) | 4 (4-5) | 3 |
| **Positive Task Self-Efficacy** | | | | | | | |
| *I am confident in my ability to use the SHRed Injuries program with my students/athletes/clients* | 0 | 0 | 2 (4) | 15 (33) | 28 (62) | 5 (4-5) | 3 |
| *I have the appropriate skills to implement the SHRed Injuries program* | 0 | 0 | 1 (2) | 15 (34) | 28 (64) | 5 (4-5) | 4 |
| **Negative Task Self-Efficacy** | | | | | | | |
| *I do not know enough about the SHRed Injuries program to implement it with my students/athletes/clients* | 27 (60) | 14 (31) | 0 | 4 (9) | 0 | 1 (1-2) | 3 |
| *I do not know how to give adequate feedback on the correct exercise techniques on the SHRed Injuries program* | 23 (51) | 11 (24) | 3 (7) | 7 (16) | 1 (2) | 1 (1-2) | 3 |
| **Maintenance Self-Efficacy** | | | | | | | |
| *I can complete the SHRed Injuries program even if my students/athletes/clients are at differing fitness levels* | 0 | 1 (2) | 0 | 14 (31) | 30 (67) | 5 (4-5) | 3 |
| *I can complete the SHRed Injuries program even if there are space limitations* | 0 | 0 | 1 (2) | 14 (3) | 30 (67) | 5 (4-5) | 3 |
| **Outcome Expectancy** | | | | | | |  |
| *I believe using the SHRed injuries program regularly will reduce the number of injuries in my students/athletes/clients* | 0 | 0 | 3 (7) | 14 (32) | 27 (61) | 5 (4-5) | 4 |
